# Expertise‐Dependent Brain Network Organization During Music Perception

**DOI:** 10.1002/hbm.70420

**Published:** 2025-11-29

**Authors:** Eleftheria Papadaki, Ziyong Lin, André Werner, Andreas M. Brandmaier, Ulman Lindenberger, Simone Kühn, Elisabeth Wenger

**Affiliations:** ^1^ Center for Lifespan Psychology, Max‐Planck Institute for Human Development Berlin Germany; ^2^ Max‐Planck UCL Centre for Computational Psychiatry and Ageing Research Berlin Germany; ^3^ Max‐Planck UCL Centre for Computational Psychiatry and Ageing Research London UK; ^4^ Department of Psychology MSB Medical School Berlin Berlin Germany; ^5^ Center for Environmental Neuroscience, Max‐Planck Institute for Human Development Berlin Germany; ^6^ Department of Psychiatry and Psychotherapy University Clinic Hamburg‐Eppendorf Hamburg Germany; ^7^ Department of Psychology HMU Health and Medical University Potsdam Germany

**Keywords:** dynamic functional connectivity, expertise, graph measures, music listening, static functional connectivity

## Abstract

Listening to music is a ubiquitous human activity, but little is known about its functional cerebral correlates. We investigated the dynamics of fMRI‐based brain activation patterns associated with two musical compositions and examined whether these patterns are modulated by the degree of musical expertise. Specifically, 24 aspiring professionals and 17 amateur musicians listened to a baroque composition by J. S. Bach and an early modern piece by A. Webern. Using measures of dynamic and static functional connectivity and graph theory, we identified two distinct brain states: one characterized by higher modularity (greater segregation), and the other by higher global efficiency (greater integration). Participants spent more time in the segregated state while listening to Bach, and more frequently shifted to the integrated state during Webern's piece. An anticorrelation was observed between segregation and music complexity as measured by permutation entropy, indicating that music with higher complexity elicited more integrated brain states. Individuals with greater musical expertise demonstrated higher global efficiency during the Webern piece and engaged more frontal, temporal, and parietal regions as functional hubs. These findings suggest that musical form and expertise jointly shape the brain's functional organization during naturalistic music listening.

## Key Points

- Dynamics of brain states are modulated by the processing requirements of music pieces.
- Greater musical expertise is associated with enhanced network integration in the listening condition with higher musical complexity.
- Greater musical expertise is associated with increased flexibility in the brain's functional organization, potentially enabling more effective adaptation to the challenges of music listening.

## Graphical abstract

Brain state dynamics adjust to the processing demands of different musical pieces, with higher expertise linked to higher network integration and flexibility in functional organization, enabling more effective adaptation to the challenges of music listening.

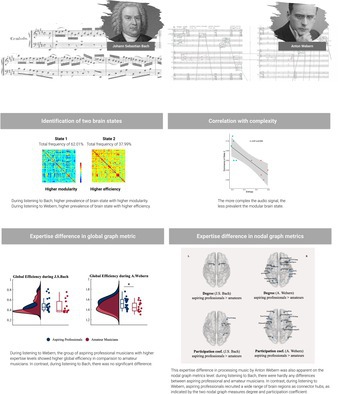

## 1 Introduction

In the past decade, neuroimaging studies on music perception have increasingly adopted the use of naturalistic stimuli in settings of continuous music listening (Alluri et al. 2017; Hoefle et al. 2018; Toiviainen et al. 2014; Wilkins et al. 2014). This shift toward naturalistic experimental paradigms resembling real‐world experiences allows for a better understanding of how the brain processes complex and ecologically valid stimuli. Some of these studies have located brain regions and extended networks of brain areas along the auditory pathways, crucial for processing timbral, rhythmic, and tonal features of musical stimuli (Alluri et al. 2012; Toiviainen et al. 2020). Through classification techniques, researchers have even been able to predict activation in regions associated with processing these acoustic features based on musical input (Alluri et al. 2013; Toiviainen et al. 2014), while distinct activation patterns can be linked to different musical pieces (Hoefle et al. 2018). Moreover, several studies have shifted focus to the cultural and extra‐musical dimensions of music perception, identifying brain regions involved in self‐referential appraisal and esthetic judgments (Alluri et al. 2013), cultural familiarity (Demorest et al. 2010) and musical preference (Bonomo et al. 2022). In a recently published study, music perceived as beautiful was found to shift brain activity from basic auditory regions to broader sensory‐motor, reward‐related, and imagery areas, enhancing integrative neural communication, while music perceived as less beautiful elicited more localized auditory activation, reflecting greater cognitive effort (Dai et al. 2024).

Building on this foundation, we add to this line of research by exploring whole‐brain functional connectivity patterns in response to naturalistic musical stimuli. Rather than concentrating on specific brain regions or acoustic features, we adopt a broader perspective, examining how music listening shapes global brain organization, using dynamic functional connectivity (DFC) and graph measures. DFC refers to the temporal variations in functional connections among different brain regions (Hutchison et al. 2013). Through advanced analytical methods, recurring connectivity patterns can be identified and organized into distinct configurations or states (Hutchison et al. 2013; Lurie et al. 2019; Preti et al. 2017). These dynamic states have been linked to various aspects of brain function across a range of paradigms, including cognitive processes (Gonzalez‐Castillo et al. 2015; Simony et al. 2016), disease states (Hutchison et al. 2013), and resting state activity (Allen et al. 2014; Calhoun et al. 2014). Additionally, graph theory provides a powerful framework for modeling the brain as a network, where nodes represent brain regions or neural assemblies and edges represent the functional connections between them (Rubinov and Sporns 2010). Within this framework, various metrics are used to characterize both local and global features of the brain's network organization, focusing primarily on two key aspects: segregation and integration (Bassett and Sporns 2017; Sporns 2013). Segregation refers to the ability of specialized brain regions to process information independently within smaller subnetworks, while integration reflects the brain's capacity to combine and share information across different regions.

In addition to characterizing the whole brain functional organization during unconstrained music listening, we aimed to investigate how varying levels of musical expertise influence this organization. There is substantial evidence that musical training has a profound modulatory effect on grey (Bermudez and Zatorre 2005; Gaser and Schlaug 2003; Palomar‐García et al. 2017; Wenger et al. 2021) and white matter structure (Abdul‐Kareem et al. 2011) and on functional activation and connectivity (Bianchi et al. 2017; Olszewska et al. 2021). In the context of unconstrained music listening, many studies have focused on activation strength, showing heightened activity in musicians, particularly in auditory cortex regions (Angulo‐Perkins et al. 2014), frontal lobe, primary and supplementary motor areas (Bangert et al. 2006; Habermeyer et al. 2009), and parietal areas (Oechslin et al. 2013; Seung et al. 2005). Beyond activation strength, musicians exhibit greater integration within music‐processing networks (González et al. 2021) and between motor and somatosensory regions (Oechslin et al. 2013). Importantly, differences in brain network organization based on musical proficiency have also been observed. During listening to music, musicians' key regions of activity (hubs) were located in cerebral and cerebellar sensorimotor areas, while nonmusicians' key hubs were found in regions associated with the default mode network (DMN) (Alluri et al. 2017). These findings indicate that musicians and nonmusicians rely on distinct brain networks when processing music, reflecting differences in their neural pathways shaped by expertise (Brattico and Delussi 2024).

In this study, we presented participants with two very different musical pieces, one composed by J. S. Bach and one by A. Webern, introducing two distinct listening conditions that likely involve different cognitive processes. The piece by J. S. Bach, an example of baroque music composition, represents a familiar and culturally ingrained musical style for Western listeners, which has been commonly used in music‐related neuroscientific research. In contrast, the piece by A. Webern, an example of the 2nd Viennese School, part of the movement of compositional innovations of the 20th/21st century, challenges listeners with its avant‐garde, atonal structure, departing from the tonal and rhythmic conventions of Western classical music (Mencke et al. 2019).

We first extracted acoustic features of the two pieces regarding tonality and rhythm, highlighting their compositional differences through established musical metrics of the MIR toolbox (Lartillot et al. 2008), following work that has been applied in contexts of both tonal and atonal music (Alluri et al. 2013, 2012; Mencke et al. 2019; Saari et al. 2018; Toiviainen et al. 2014). We also computed the permutation entropy of each piece, a measure of complexity and unpredictability in time series, to further differentiate them. This quantification allows us to showcase the distinct cognitive demands each piece places on the listener.

Next, we examined how these listening conditions related to brain connectivity dynamics by conducting a DFC analysis. To further quantify these dynamics, we utilized a state metric that captures the average number of time windows a participant spent in each state. We employed two graph metrics—modularity and global efficiency—to describe the network configurations that emerged from the DFC analysis. Modularity, a measure of segregation, assesses how well a network segregates into communities, that is, clusters of densely interconnected regions with sparse connections to other clusters (Sporns and Betzel 2016). Global efficiency, on the other hand, is a measure of network integration indicative of the ease of communication efficacy among regions of a network (Rubinov and Sporns 2010).

We hypothesized that (i) listening to Webern's piece with its unfamiliar and intricate structure would result in brain states characterized by higher global integration and reduced modularity compared to Bach's piece. This prediction is based on prior findings relating cognitive demands to whole‐brain states. Emergence of more integrated, globally efficient and less clustered states has been observed in cognitively effortful tasks and is hypothesized to facilitate adaptability and performance (Kitzbichler et al. 2011; Shine et al. 2016; Vatansever et al. 2015). The reversed pattern of brain states with higher modularity has been observed for tasks tapping into more automatic/habitual processing (Shine and Poldrack 2018). This relationship between states of higher integration for more complex input should not only hold between the two music pieces but also within each of the pieces. We therefore further hypothesized that (ii) within each piece, higher levels of complexity, as measured by permutation entropy, would correlate with more integrated brain states.

With respect to the effect of musical expertise on unconstrained music listening, we applied static FC analysis and computed the graph measure of global efficiency to investigate group differences in brain network architecture. We expected that (iii) the group of higher expertise would demonstrate higher global efficiency, particularly during the Webern piece, reflecting more effective information integration and potentially more skillful cognitive processing. To further explore the functional roles of specific brain regions, we also computed two nodal graph measures: nodal degree and participation coefficient. Nodal degree indicates which brain regions act as central hubs, facilitating information flow and occupying key positions in the brain's functional network (Power et al. 2013; van den Heuvel and Sporns 2013). The participation coefficient highlights regions that connect different subnetworks, acting as bridges between modular communities (van den Heuvel and Sporns 2013). We hypothesized that (iv) the group with higher expertise would exhibit higher nodal degree and participation coefficient across a range of brain regions critical for music processing, particularly in the condition of listening to A. Webern. This would suggest that their training enables them to better recruit and utilize the available functional repertoire to meet the demands of different listening conditions.

## 2 Materials and Methods

### 2.1 Participants

In our analysis, we used a subset of data from the PITCH study, a longitudinal study assessing structural and functional brain changes in the course of musical training. A full description of the study and other findings using this sample on gray matter volume changes and expertise differences in functional connectivity during interval recognition are reported, respectively, by Wenger et al. (2021) and Papadaki et al. (2023). We repeat all relevant details regarding subject recruitment, study design, and acquired measures for the current analyses here.

Forty‐one participants (*M*_age_ = 22.35, SD = 3.63, 15 female) were recruited through flyers, mailing lists, and word‐of‐mouth in Berlin, Germany. Of these, 24 were aspiring professional musicians enrolled in preparatory courses at Berlin music schools, where they received comprehensive training in music theory, ear training, history, and ensemble playing. The other 17 were amateur musicians, performing music regularly but without professional aspirations. All participants had at least 5 years of experience with their primary instrument or vocals, with comparable years of practice between groups *t*(38) < 1, *p* = 0.68 (amateur musicians: *M*_years_ = 12.74, SD = 5.97; aspiring professional musicians: *M*_years_ = 12.04, SD = 4.56; one participant in the aspiring professional group did not provide information about his or her primary instrument or years of playing). Importantly, aspiring professionals practiced daily on their primary instrument/vocals significantly more, *t*(39) = 3.7, *p* = 0.001, Cohen's *d* = 1.2 (amateur musicians: *M*_hours_ = 1.2, SD = 0.8; aspiring professional musicians: *M*_hours_ = 2.6, SD = 1.4) and dedicated more time to music theory learning *t*(39) = 4.91, *p* = 0.001, Cohen's *d* = 2.5 (amateur musicians: *M*_hours_ = 0.2, SD = 0.3; aspiring professional musicians: *M*_hours_ = 1.4, SD = 0.6) (see Table 1). They also scored higher in a separate behavioral measure of musical expertise, the Berlin Gehoerbildung Scale (BGS; Lin et al. 2021), *t*(39) = 5.72, *p* < 0.001, Cohen's *d* = 1.8 (amateur musicians: *M* = −0.56, SD = 0.46; aspiring professional musicians: *M* = 0.4, SD = 0.65). The scale comprises a set of listening and transcription tasks within the tradition of western art music, requiring formal music education and training.

**TABLE 1:** Summary table of sample characteristics regarding age, years of engagement with primary instrument or voice training, daily amount of primary instrument practice, daily amount of music theoretical learning, and handedness (for five participants, there is no report on handedness).

	Age (years)		Music learning primary instrument: voice (years)		Instrument practice (daily hours)		Music theory learning (daily hours)		Handedness	
	*M*	SD	*M*	SD	*M*	SD	*M*	SD	Left	Right
Aspiring professionals	21.92	3.72	12.04	4.56	2.6	1.4	1.4	0.6	1	20
Amateur musicians	23	3.5	12.04	5.97	1.2	0.8	0.2	0.3	1	14

Therefore, our sample comprises two groups of people who have been musically engaged for approximately the same amount of time. A decisive difference lies in the intensity of the training given the different intentions in their musical practice, with aspiring professional musicians undergoing intensive, both practical and theoretical, learning with their respective musical instruments in order to be accepted to music university programs. Therefore, the different levels of expertise are characterized not simply by the amount of time of engagement with music but rather by the intensity of this engagement and the motivation behind it. Participants of both groups did not differ significantly with respect to age, *t*(39) < −1.05, *p* = 0.30 (amateur musicians: *M*_age_ = 23.00, SD = 3.50, 8 female; aspiring professional musicians: *M*_*age*_ = 21.92, SD = 3.72, 7 female). Regarding handedness, 33 participants were right‐handed, 2 were left‐handed (1 in the group of aspiring professionals and 1 in the group of amateur musicians), and for 5 participants (3 in the group of aspiring professionals and 2 in the group of amateur musicians) there was no report on their handedness. All participants had normal hearing, did not have any metallic implants, and had not had any psychiatric diagnosis.

Participants were paid up to 200€ for completion of the whole study (including up to five measurement time points with 1.5 h of MRI and 1.5 h of behavioral testing). The ethical board of the DGPs (Ethikkommission der Deutschen Gesellschaft für Psychologie) approved the study, and written consent of all participants was obtained prior to the investigation.

### 2.2 fMRI Task: Unconstrained Music Listening

Participants underwent two listening conditions during the fMRI data acquisition. In the first condition, they listened to Johann Sebastian Bach's Harpsichord Concerto in E major, BWV. 1053 Allegro, bars 1–321, a piece of the baroque music genre, for a duration of 5 min. In the second condition, they listened to a piece by Anton Webern, namely Variations Op. 30, bars 1–134, a piece belonging to the Second Viennese School, likewise for a duration of 5 min. We would like to emphasize that the more general labeling of those two specific pieces as “baroque” versus “Second Viennese School” is simply done to ease the understanding and is not meant as an indication for broader generalizability.

Both musical pieces present distinct compositional and stylistic features that make them valuable for investigating brain dynamics during music listening. Anton Webern's Variationen für Orchester, Op. 30 (1940/41) is his penultimate completed work. The piece is built on a symmetrical 12‐tone row—its first six notes followed by the retrograde inversion—containing internally symmetrical four‐note segments. This row serves as the foundation for both the pitch development and the overall formal structure through continuous variation. Rhythmically, the composition features frequent time signature changes that produce a fragmented, pointillistic texture. The third movement (Allegro) of J. S. Bach's Concerto in E major, BWV 1053 (1738), is structured in a da capo A–B–A form and demonstrates Bach's mastery through subtle rhythmic and harmonic complexity. Though rhythmically animated, it is grounded in a stable tonal and metrical framework. Ambiguities like the opening triplet figures or chromatic sequences momentarily blur pulse and tonality, but eventually are resolved in the context of its formal and tonal structure.

### 2.3 fMRI Data Acquisition

MR images were acquired on a 3 T Siemens Tim Trio scanner with a 12‐channel head coil. Structural images used a 3D T1‐weighted MPRAGE sequence (9.20 min): TR = 2500 ms, TE = 4.77 ms, TI = 1100 ms, flip angle = 7°, bandwidth = 140 Hz/pixel, acquisition matrix = 256 × 256 × 192, and voxel size = 1 mm^3^. Pre‐scan normalization and 3D distortion correction were applied. Functional images were collected with two 5‐min T2*‐weighted EPI sequences: TR = 2000 ms, TE = 30 ms, FOV = 216 × 216 × 129 mm^3^, flip angle = 80°, slice thickness = 3.0 mm, distance factor = 20%, voxel size = 3 mm^3^, 36 axial slices, and GRAPPA acceleration (Factor 2). Slices were acquired in an interleaved fashion and aligned to the corpus callosum.

### 2.4 fMRI Preprocessing

The fMRI data were preprocessed using DPABI V4.3 (Yan et al. 2016), under MATLAB 2018a (The MathWorks). The first 10 time points were discarded for signal stabilization. Remaining volumes were corrected for acquisition time differences, realigned, and co‐registered to structural images, which were segmented into GM, WM, and CSF (Ashburner and Friston 2005). Nuisance signals (head motion, WM, CSF, respiratory/cardiac effects) were regressed out using the Friston 24‐parameter model (Friston et al. 1996), alongside linear and quadratic trends. Images were normalized to MNI space (3 × 3 × 3 mm), spatially smoothed (4‐mm FWHM), and temporally filtered (0.01–0.1 Hz).

### 2.5 Musical Feature and Entropy Analysis

Musical feature analysis was conducted using the MIR toolbox (v1.8.1) (Lartillot et al. 2008; Lartillot and Toiviainen 2007) in MATLAB 2019b. This toolbox extracts features from audio files related to dynamics, timbre, pitch, tonality, and rhythm, which have been validated via a perceptual experiment (Alluri et al. 2012). For our analysis, we focused on tonality (key clarity and chromagram) and rhythm (pulse clarity) to provide a descriptive overview of the two musical pieces. Of course, the features extracted are by no means exhaustive of what constitutes the whole experience of listening to these musical pieces. They highlight some points of difference between the two musical pieces, facilitating the generation of hypotheses regarding differences in the functional organization of brain states while listening to the two musical pieces. Regarding the tonality features, key clarity gives an estimation of the presence of each key in the signal at any given moment and is calculated based on the pitch chromagram and the Krumhansl–Kessler algorithm, matching pitch class profiles to key profiles (Krumhansl 2001; Toiviainen and Krumhansl 2003), using a window size of 5 s and a hop factor of 33%, following Lartillot and Toiviainen (2007). The chromagram, or also harmonic piece class profile, shows the distribution of energy along pitch classes and is computed on a logarithmic scale using the fast Fourier transformation (FFT). It is computed using a frame length of 200 ms and a hop factor of 5% (10 ms) (Lartillot and Toiviainen 2007). From the rhythmic features, pulse clarity gives an estimation of rhythmic clarity, indicating the strength of beats. It is computed using the maximum correlation value in the autocorrelation curve as a heuristic (Lartillot et al. 2008), using again a window size of 5 s and a hop factor of 33. Further information regarding the aforementioned terms and procedures can be found in Supporting Information S1.

In addition, we computed the permutation entropy as a measure of complexity and irregularity of the time series of the two music pieces using the pdc R package (Brandmaier 2015). The time series of each piece was chunked into five segments of 60 s, and the permutation distribution was computed for each segment, assigning a probability to the occurrence of certain order patterns. The order patterns were computed with an embedding dimension of 7. Then the entropy over the distribution of observed order patterns was computed for each segment (Bandt and Pompe 2002). Maximum entropy corresponds to a white noise signal, whereas lower entropy indicates higher predictability of the time series.

### 2.6 Assessing Network Differences Between Listening Conditions

#### 2.6.1 DFC Analysis

After preprocessing the data, we extracted the time courses of 112 brain regions taken from the Harvard–Oxford atlas (Desikan et al. 2006) for each participant and during both listening conditions, using DPABI V4.3. These were further analyzed with the DynamicBC toolbox (Liao et al. 2014) under MATLAB 2019b, computing the DFC using a sliding window approach, with a window length of 60 s moving in steps of 1 TR (2 s), *across both musical pieces*. Further, the DFC matrices of all subjects underwent k‐means clustering analysis, which collapses the temporal dimension of dynamic connectivity matrices into several connectivity maps describing the recurring patterns of activation during listening to the musical pieces (Allen et al. 2014; Liu and Duyn 2013)—referred to from here on as states. The optimal number of cluster partitions was 2 and was based on the convergence of four distance measures: Squared Euclidean distance (*k* = 2), Silhouette index (*k* = 2), Calinski–Harabasz index (*k* = 2), and Davies–Bouldin index (*k* = 2).

#### 2.6.2 Graph Measures and State Metric on DFC Analysis

We sought to characterize each state in terms of segregation and integration by computing the graph‐theoretic measures *modularity index* and *global efficiency*, using the Brain Connectivity Toolbox (https://sites.google.com/site/bctnet/; Rubinov and Sporns 2010). Modularity measures the extent to which a network can be divided into communities, maximizing within‐community edges and minimizing between‐community edges. It is computed using a fast multi‐iterative generalization of the Louvain community detection algorithm (Blondel et al. 2008; Newman 2006; Reichardt and Bornholdt 2006). Global efficiency is a measure of information transmission between the nodes of the network. At the nodal level, it characterizes the efficiency of information transfer from one region to the whole network and is computed as the inverse of the average shortest path length between one node and all other nodes in the network. Global efficiency at the global level is the average of the global efficiency of all nodes in the graph and is inversely related to the characteristic path length (Latora and Marchiori 2001). After setting negative correlations to 0—as is common practice in order to compute modularity, modularity and global efficiency were calculated for each subject's connectivity matrices across all windows within each state and averaged per subject. Subsequently, two‐way analyses of variance (ANOVA) assessed the effects of listening condition, state, and their interaction on these measures, followed by post hoc Tukey–Kramer tests for unequal sample sizes, since each participant was included in the statistical tests only for the states they actually found themselves in.

Furthermore, the commonly used metric of frequency was calculated to assess the participation of individuals in the states across listening conditions. Frequency is computed as the average number of time windows a participant spent in a state, expressed as a fraction. Differences in frequency between listening conditions were computed using paired‐sample *t*‐tests. To increase the robustness of our findings, we repeated the analysis using the Dosenbach 160 atlas (Dosenbach et al. 2010), and the results provided support for the consistency of our results across different parcellation schemes (see Supporting Information S1).

#### 2.6.3 Brain States and Entropy of the Music Pieces

Finally, we looked into the relationship of brain states with the entropy of the time series of the music pieces, in order to assess whether the complexity of the audio signal relates to observed brain states. For each of the five segments of 60 s of the musical pieces, the entropy value was correlated using Pearson's *r* correlation coefficient with the occurrence of brain states, namely, which state was dominating in each time segment.

### 2.7 Assessing Expertise‐Related Differences in Music Listening

#### 2.7.1 Graph Theory Analysis of Static FC

We used a static FC analysis for each listening condition separately and examined expertise differences in global (i.e., whole‐brain) and nodal graph measures. Using these nodal graph measures, we aimed to zoom in on specific regions that act as hubs, occupying a central position in network organization, and as connector hubs, assisting between modules, and demonstrate expertise differences therein.

#### 2.7.2 Network Construction

The network construction and graph analyses were carried out using the Brain Connectivity Toolbox (https://sites.google.com/site/bctnet/; Rubinov and Sporns 2010). Time courses were extracted for each of the 112 regions of the Harvard–Oxford atlas (Desikan et al. 2006), and Pearson's *r* correlation coefficient was computed on the time courses for each pair of regions (ROIs), resulting in a 112 × 112 correlation matrix. Following Bassett and Gazzaniga (2011), *t*‐tests were calculated on the correlation coefficients for each pair of ROIs in the connectivity matrix of each participant, and FDR correction was applied to the *p* values, such that only those correlations were retained that remained significant, resulting in weighted, undirected connectivity matrices. Negative weights were again converted to zeros, a prerequisite to computing graph measures like modularity.

#### 2.7.3 Network Analysis

To characterize expertise‐related differences in the whole brain, we chose the metric of global efficiency. As described above, global efficiency is a measure of information transmission between the nodes of the network. Expertise differences for both conditions were assessed using two‐sample *t*‐tests. Additionally, for the Bach condition, expertise differences were assessed using a Mann–Whitney *U*‐test for independent samples, as the values were not normally distributed. To increase the robustness of our findings, we repeated the analysis using the Dosenbach 160 atlas (Dosenbach et al. 2010), and the results provided support for the consistency of our results across different parcellation schemes (see Supporting Information S1).

In addition, we computed two nodal metrics, *degree* and *participation coefficient*, for each participant in each listening condition to assess hubs and connector hubs. The degree or degree centrality refers to the number of edges connected to a specific node. The weights of the connections were ignored by binarizing the connectivity matrix so that only edges with nonzero weights were considered connected. Degree is considered a proxy of the centrality of a node, indicating its importance within its community (provincial hub), connecting primarily with nodes within its community (van den Heuvel and Sporns 2013). Participation coefficient measures how much a node displays a diverse connectivity profile, communicating with nodes of different modules/communities. Nodes with high participation coefficients are thought of as connector nodes, potentially transmitting information between modules (van den Heuvel and Sporns 2013). Participation coefficient measures the uniformity of the distribution of connections of a node to nodes from all community partitions (Guimerà and Nunes Amaral 2005) and is computed following the calculation of community structure (Blondel et al. 2008; Reichardt and Bornholdt 2006).

For each subject, we computed one‐sample *t*‐tests on the measures of nodal degree and participation coefficient for each node and subsequently corrected for multiple testing using FDR correction. We tested for expertise differences for each listening condition separately using two‐sample *t*‐tests on the degree and participation coefficient for the nodes surviving statistical testing and FDR correction. Additionally, we tested for differences in degree between the two listening conditions for each group, using paired‐sample *t*‐tests, to assess how groups of different expertise shift their hub profiles based on condition demands.

## 3 Results

### 3.1 Musical Feature and Entropy Analysis

**FIGURE 1:**
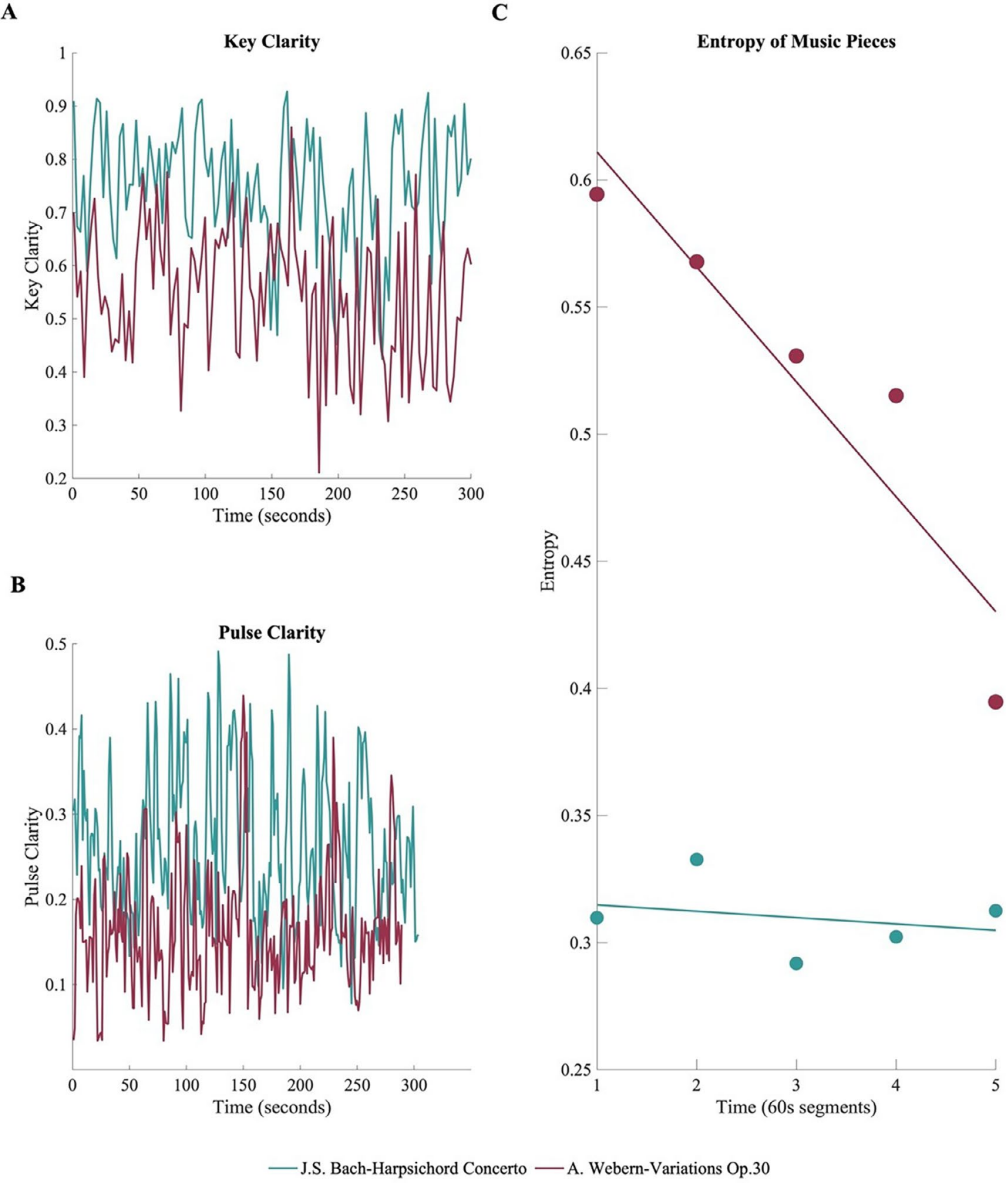
Musical features and entropy analysis. (A) and (B) Key clarity and pulse clarity estimated for both pieces, with overall higher values for the piece by Bach. (C) Entropy values for each 60‐s segment computed on the permutation distribution of the time series of the two musical pieces. The piece by Webern was characterized by higher entropy than the piece by Bach across all time segments.

Analysis of the two musical pieces entailed the extraction of tonality and rhythmic features, providing a quantitative description of key musical characteristics influencing the listening process. As shown in Figure 1A, key clarity differed between the two pieces with the piece by Bach exhibiting a higher mean value (*M* = 0.745, SD = 0.115) than the piece by Webern (*M* = 0.546, SD = 0.12). As for rhythmic features, the piece by Bach exhibited a higher mean value of pulse clarity (*M* = 0.264, SD = 0.08) than the piece by Webern (*M* = 0.161, SD = 0.07; Figure 1B).

The chromagram (see Figure S1) clearly showed differences between the two pieces: there was more variation in the distribution of energy along pitch classes in the piece by Bach, while in the piece by Webern, the pitch classes were more equally represented, as expected in the composition style that does not follow tonal hierarchies.

Finally, the computation of permutation entropy of the time series of the two pieces showed that the piece by Webern had higher entropy, indicating higher complexity, than the piece by Bach for all time segments (Figure 1C). The numeric entropy estimates for each 60 s segment of each piece can be found in Table S1.

### 3.2 Network Differences Between Listening Conditions

#### 3.2.1 During Both Music Pieces, Two Brain States Emerged: One With Higher Integration, One With Higher Modularity

**FIGURE 2:**
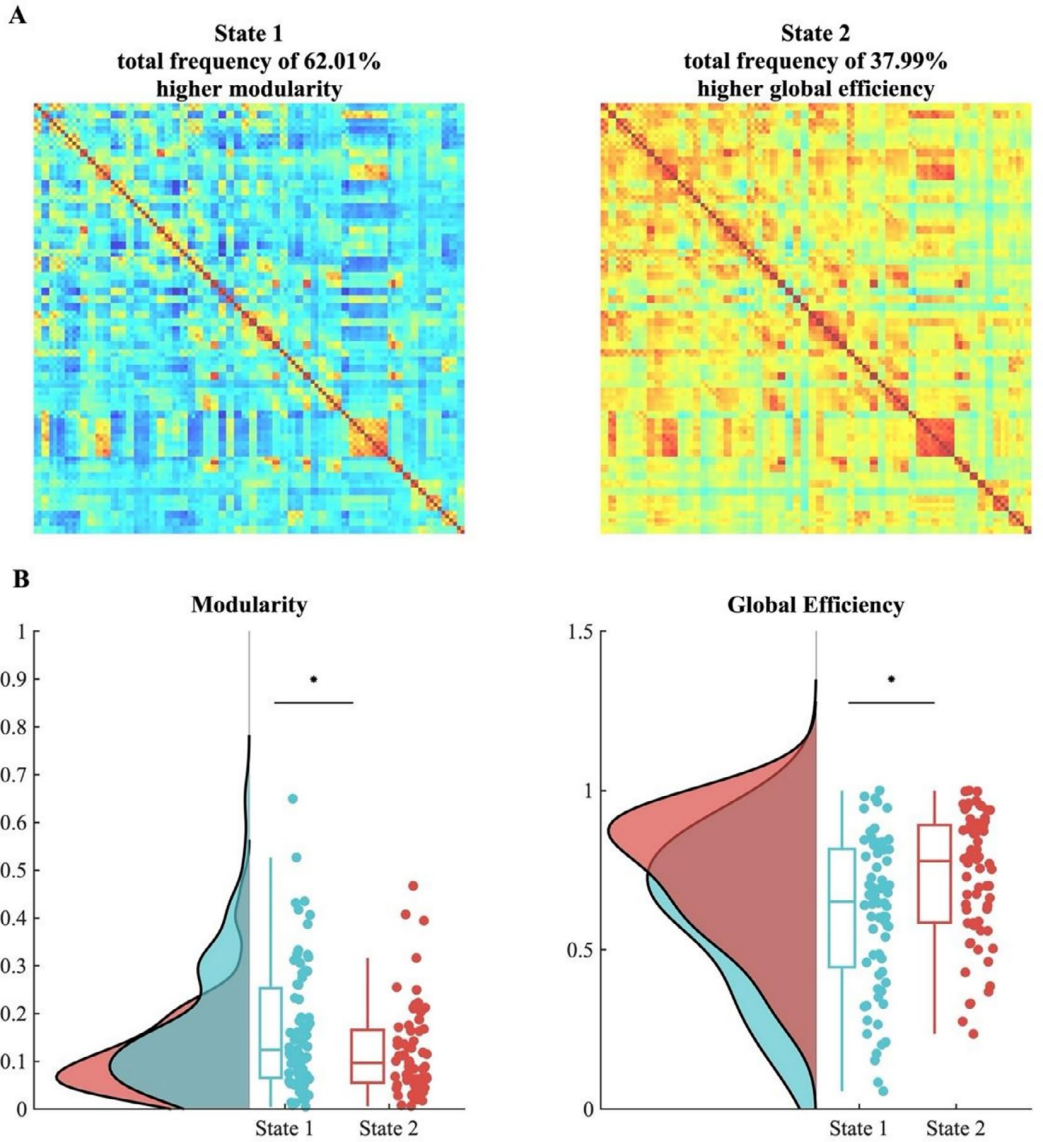
Brain states and network differences. (A) Brain states detected with DFC analysis. The matrices represent the 112 ROIs from the Harvard–Oxford atlas and are uncorrected and displayed for visualization purposes. (B) Graph measures computed on the brain states across both listening conditions. The two states differed in modularity and global efficiency, with the first state exhibiting significantly higher modularity and the second state exhibiting significantly higher global efficiency, indicative of higher overall integration. Graph measures distributions are shown as raincloud plots (Allen et al. 2019) and boxplots with medians and 95% CI with whiskers representing the 2nd and 98th percentiles. Each dot represents a single subject. Asterisks indicate a significant effect at *p* < 0.05. Exclusion of individual values in the modularity index that exceeded 2 SDs still resulted in a significant difference at *p* = 0.04.

The results of the DFC analysis and the k‐means clustering identified the two most prominent states across listening conditions. One occurred at a mean frequency of 62.01% and the other at a mean frequency of 37.99% (Figure 2A). In order to characterize each state in terms of segregation and integration, we computed the modularity and global efficiency for each subject's connectivity matrices of all windows and averaged for each subject state‐wise. The two states differed significantly in terms of modularity (*F*(1,1) = 5.98, *p* = 0.015) and global efficiency (*F*(1,1) = 3.72, *p* = 0.05; see Figure 2B). Post hoc Tukey–Kramer tests showed that higher modularity values were prevalent in State 1 (*t* = 2.446 *p* = 0.014, Cohen's *d* = 0.4; State 1: *M* = 0.17, SD = 0.15; State 2: *M* = 0.12, SD = 0.09) and higher global efficiency was prevalent in State 2 (*t* = 3.0428 *p* = 0.0028, Cohen's *d* = 0.56; State 1: *M* = 0.61, SD = 0.23; State 2: *M* = 0.7323, SD = 0.2), and this was true across listening conditions.

#### 3.2.2 During Listening to Bach, Higher Prevalence of Brain State With Higher Modularity. During Listening to Webern, Higher Prevalence of Brain State With Higher Integration

**FIGURE 3:**
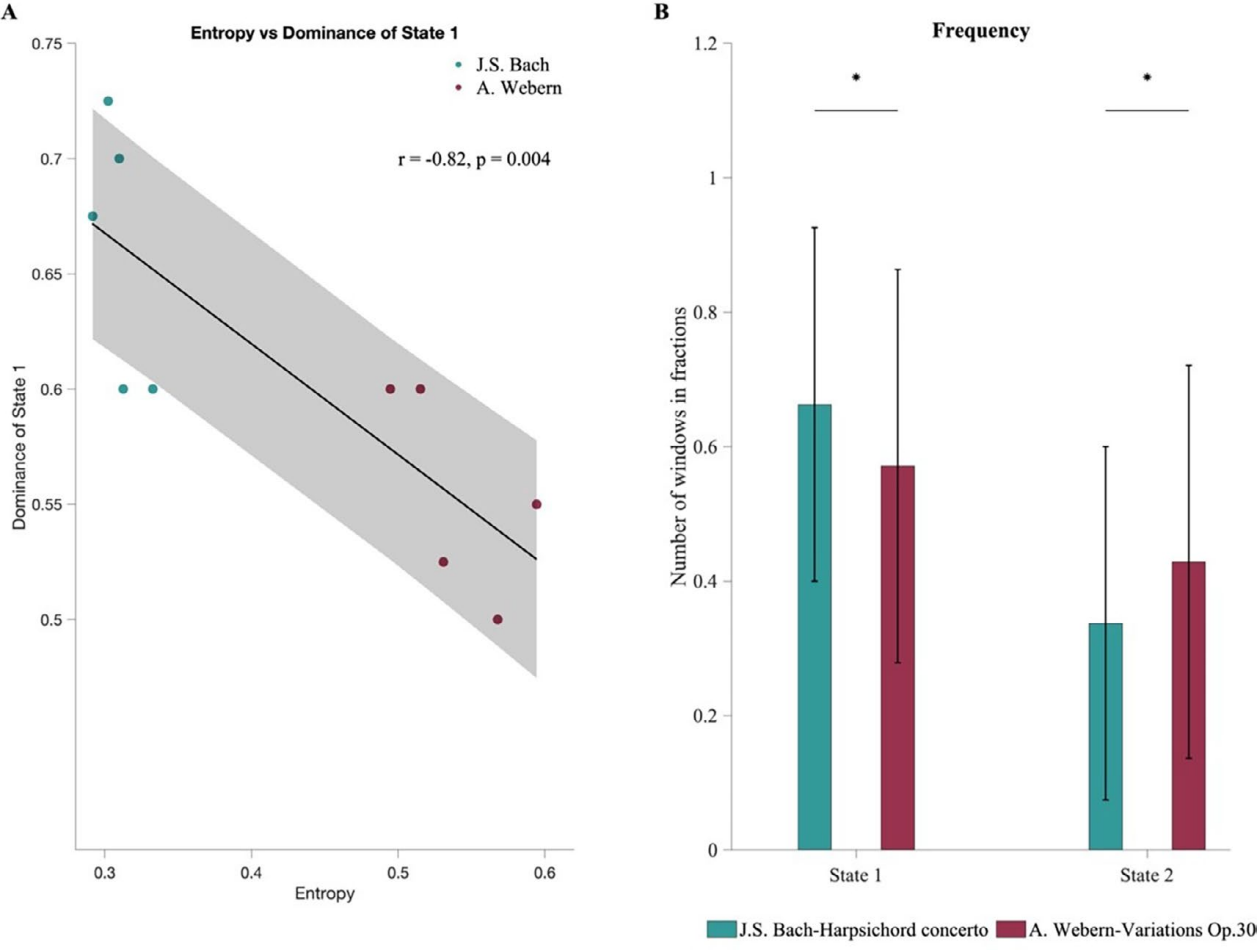
Relationship between brain states, musical complexity, and listening conditions. (A) Correlation of brain state occurrence with the entropy of the musical time series. A strong anticorrelation was observed between the occurrence of the more modular brain state and the entropy of the musical pieces, reflecting signal complexity (*r* = −0.81, *p* = 0.004). In time segments of increased complexity, participants were less likely to remain in the modular brain state, favoring a more integrated state. (B) State metric of frequency computed on the dynamic functional connectivity analysis across both listening conditions. During listening to the piece by Bach, participants spent significantly more time in the more modular state, while they spent on average more time in the more integrated state when listening to Webern. Whiskers in the state metrics indicate standard deviation. Asterisks indicate significant effect at *p* < 0.05.

We used a paired *t*‐test to compute differences between the two listening conditions for the state metric of frequency, which is the expression in fractions of the time windows participants spent in each state. Frequency differed significantly between the two listening conditions, with participants spending more time in the first state of higher modularity during listening to Bach (*t*(39) = 1.954, *p* = 0.05, Cohen's *d* = 1.06) and in the second state of higher integration during listening to Webern (*t*(39) = 1.954, *p* = 0.05, Cohen's *d* = 0.75; see Figure 3B and Table S2).

#### 3.2.3 The More Complex the Audio Signal, the Less Prevalent the Modular Brain State

In order to directly assess whether the complexity of the audio signal relates to observed brain states, state occurrence in each time segment of 60 s was correlated with the entropy value of this segment of the music pieces using Pearson's *r* correlation coefficient. Indeed, we found a strong anticorrelation between the occurrence of State 1, the more modular state, and entropy (*r* = −0.81, *p* = 0.004; see Figure 3A). That is, the more complex the incoming audio signal, the less frequently participants find themselves in the state of higher modularity, but rather, per definition, in the second state of higher integration.

### 3.3 Expertise‐Related Differences in Music Listening

#### 3.3.1 Significant Expertise Differences in Global Efficiency During Listening to Webern, but Not During Bach

**FIGURE 4:**
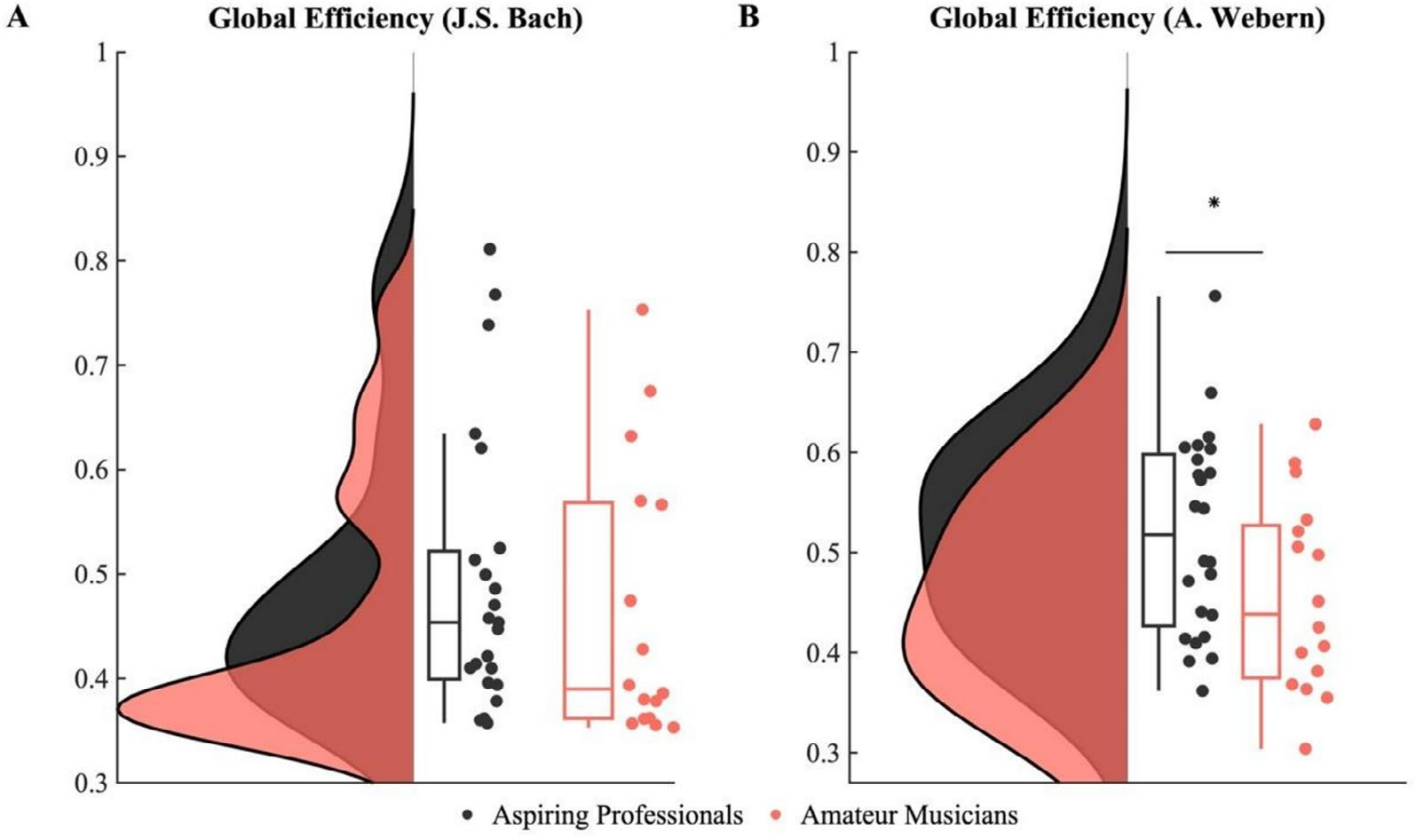
Group differences in global efficiency for the two listening conditions. (A) During listening to Bach, there were no group differences in global efficiency. (B) During listening to Webern, however, the group of aspiring professionals showed significantly greater global efficiency than amateur musicians. Group distributions are shown as raincloud plots (Allen et al. 2019) and as boxplots with medians and 95% CI with whiskers representing the 2nd and 98th percentiles. Each dot represents a single subject. Asterisks indicate a significant group difference at *p* ≤ 0.05.

To assess expertise‐related differences in music listening, we performed a static functional connectivity analysis for each participant in each listening condition, and then computed global efficiency as a global graph measure. During listening to Bach, there were no significant group differences in global efficiency (Mann–Whitney *U*‐test = 140, *p* = 0.15, Cohen's *d* = 0.2) (Figure 4A). In contrast, during listening to Webern, we found a significant expertise difference in global efficiency (*t*(38) = 1.9451, *p* = 0.05, Cohen's *d* = 0.62) (Figure 4B).

#### 3.3.2 Group of Higher Expertise Utilized a Wide Range of Brain Regions as Hubs and Connector Hubs During Listening to Webern and Switched to a Subset of Those Regions During Listening to Bach

**FIGURE 5:**
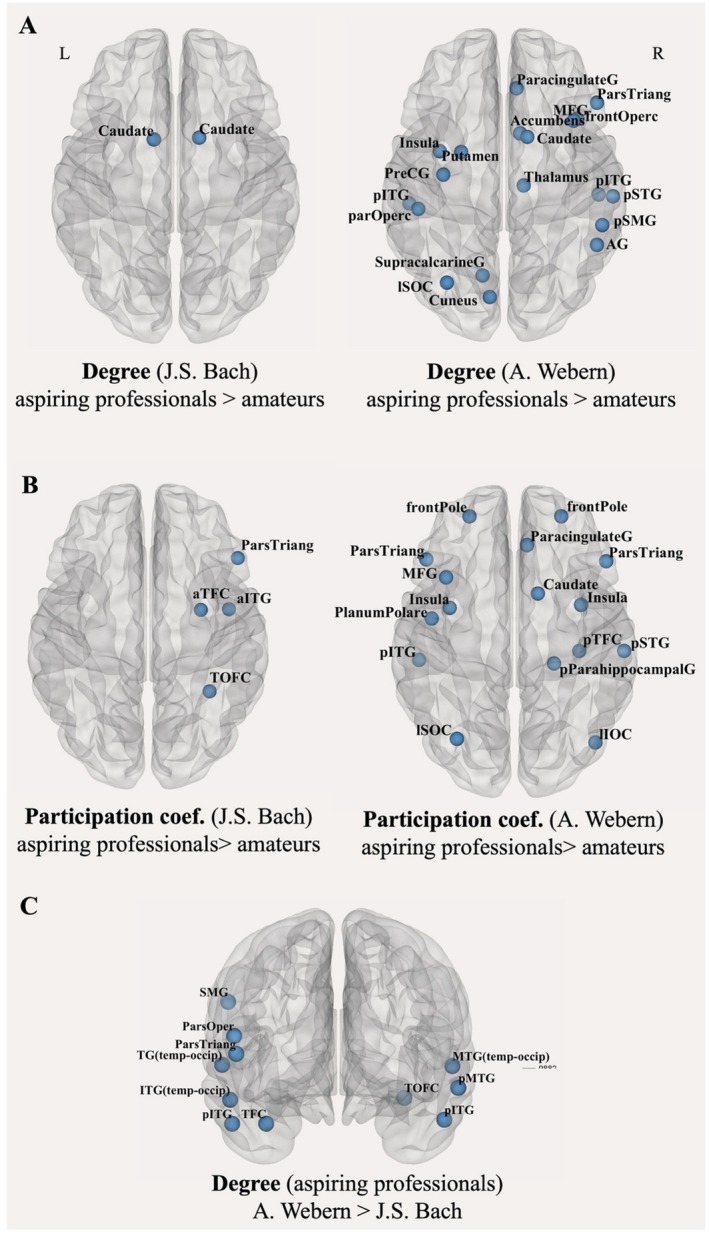
Group differences in nodal measures of degree and participation coefficient. (A) Aspiring professional musicians exhibited higher nodal degree values compared to amateur musicians, particularly during listening to Webern, indicating greater overall network connectivity in this group. (B) Aspiring professionals also showed elevated participation coefficient values, especially during the Webern piece, reflecting greater integration across brain networks. (C) Brain regions with significant differences in degree between listening conditions within the aspiring professionals group. These regions showed higher degree values during the Webern piece, indicating increased connectivity in specific areas. Results are reported at a threshold of *p* < 0.05, following FDR correction.

To further explore how musical expertise modulates the functional roles of specific brain regions, we computed the nodal graph measures of degree as an indicator of nodes acting as hubs, and participation coefficient as an indicator of nodes facilitating communication between communities. During listening to the piece by Bach, there were very few regions showing expertise‐related differences: two regions for the measure of degree, namely in the left and right caudate and four regions for the measure of participation coefficient, namely the bilateral inferior frontal gyrus, the anterior part of the right temporal fusiform gyrus and the right temporal occipital fusiform gyrus (see Figure 5 and Table S3). During listening to Webern, however, we found significant expertise‐related differences for both measures in an extended set of regions throughout the brain, namely regions of the temporal lobe like the superior temporal gyrus (STG), the inferior temporal gyrus (ITG), the middle temporal gyrus (MTG), and the planum polare, frontal and parietal regions like the inferior and middle frontal gyrus, the frontal pole, the parietal and frontal operculum and the insula, lateral occipital cortex, as well as nucleus accumbens (see Figure 5 and Table S3). In all these regions, only the group of aspiring professionals exhibited higher values. Furthermore, paired *t*‐tests for within‐group differences across listening conditions confirmed that indeed aspiring professional musicians adapted their listening network depending on differing input: we detected regions that differed significantly in the measure of degree between listening to Bach and listening to Webern in the group of aspiring professional musicians, with higher degree occurring during listening to Webern (see Figure 5C and Table S3). No such condition differences were visible in the group of amateur musicians. To ensure that group differences in graph metrics are not driven by differences in edge count, we tested for edge density differences across groups in both listening conditions and found no significant group effects.

In sum, regarding expertise‐related differences, we found no expertise‐related differences in the processing of the supposedly less demanding musical piece by Bach, but we found higher global efficiency in aspiring professional musicians during listening to Webern. In addition, aspiring professionals utilized a wide range of brain regions as hubs and connector hubs during the demanding listening condition and switched to a subset of those regions during the less demanding listening condition.

## 4 Discussion

This study aimed to identify and characterize prominent brain states during unconstrained music listening and to explore how these states are influenced by musical expertise. We identified and characterized prominent brain states during unconstrained music listening. Participants listened to a piece by J. S. Bach, representative of Baroque music, and a piece by A. Webern, reflecting the 20th‐century compositional innovations of the 2nd Viennese School. The pieces differed in metrics of tonality, rhythm, and entropy, suggestive of distinct perceptual and cognitive demands during listening. Through DFC analysis and graph‐theoretical measures, we explored differences in functional brain organization between the two listening conditions and across expertise groups. Two distinct brain states emerged: one with higher global efficiency, indicating greater integration, and another with higher modularity. Participants spent more time in the modular state while listening to Bach and more time in the integrated state during Webern. A strong anticorrelation between the state of higher modularity and entropy supported this: the more complex the audio signal, the more time participants spent in the integrated state. Next, we examined how musical expertise influenced brain network organization. Graph‐theoretic analysis of static functional connectivity revealed no expertise‐related differences for the Bach piece, but aspiring professional musicians exhibited higher global efficiency during Webern, suggesting greater adaptability to the more complex condition. This group also displayed a broader range of brain regions as hubs and connector hubs during Webern, which narrowed to a subset during Bach, reflecting an adaptation to the lower cognitive demands of the Baroque piece. In the following, we will discuss each of these findings consecutively, in more detail.

We initially used music information retrieval (MIR) methods to analyze the musical pieces, focusing on their tonal and rhythmic characteristics. Bach's piece demonstrated higher clarity in key and pulse. The observed differences in key and pulse clarity in the MIR analysis may also be attributed to the contrasting compositional features of the two pieces: Bach's piece features clearer tonal and rhythmic hierarchies, while Webern's piece treats all 12 tones more equally and incorporates more complex rhythmic structures. Tonality differences were also evident in the chromagram of the two pieces, as Bach's composition exhibited an uneven distribution of pitches along the chromatic scale, while Webern's piece displayed a more uniform chromatic pitch distribution. The two pieces also differed in permutation entropy, with Webern's work showing higher values across all time segments compared to Bach's. Permutation entropy, as computed in this study, quantifies the complexity and irregularity of the time series for each musical piece. A higher value indicates greater unpredictability in the sequence of events, suggesting a richer variety of possible patterns. However, this measure should be interpreted cautiously, as it is derived from the amplitude values of the time series, capturing irregularities in amplitude variations over time. Amplitude fluctuations, apart from their predominant role in the perception of loudness, have also been shown to influence the perception of duration (Dawson et al. 2017), to assist melodic contour identification (Luo et al. 2019), to affect the perception of rhythm (Daikoku and Goswami 2022; Patel 2003), and of timbre (Patil et al. 2012).

These two pieces evoke markedly different listening experiences. Bach's music is generally more familiar and culturally ingrained in Western audiences, adhering to tonal and metrical hierarchies that enhance predictive processing and elicit emotional responses, reward, and pleasure related to musical stimuli (Koelsch et al. 2019; Mencke et al. 2019). In contrast, Webern's music presents a challenging auditory experience; the absence of tonal and metrical hierarchies complicates cognitive processing and disrupts grouping mechanisms, which are essential for forming predictions (Rosch 1975; Mencke et al. 2019). Atonal music is characterized by high entropy and low information content (Dean and Pearce 2016), leading to experiences of “predictive uncertainty” (Hansen and Pearce 2014), complexity, and ambiguity (Mencke et al. 2019). Interestingly, recent findings suggest that the brain can predict and process even highly uncertain, atonal music through core mechanisms like cortico‐hippocampal interactions, highlighting the adaptability of perception and memory in making sense of unpredictable sound environments (Brattico and Delussi 2024). Such attributes may adversely affect performance across various perceptual and cognitive tasks, including melody recollection, transposition, processing speed, pitch detection, and expectation generation (Mencke et al. 2019, 2021). Nevertheless, or maybe even more so, contemporary‐classical music such as Webern's can evoke esthetic experiences shaped by familiarity and exposure (Dean and Pearce 2016; Omigie et al. 2017), as well as factors like “esthetic framing” (Brattico et al. 2013), openness to experience (Nusbaum and Silvia 2011), cognitive mastering (Leder et al. 2004), and processing fluency (Reber et al. 2004). Furthermore, it has the potential to evoke pleasure and reward sensations resulting from decoding perceptual uncertainties, stimulating curiosity and exploration of novel acoustic experiences (Gold et al. 2019; Mencke et al. 2019, 2021).

In our analysis of DFC, we identified two distinct brain states associated with processing the musical pieces. One state, occurring approximately 62% of the time, exhibited higher modularity, reflecting increased segregation. The other state, occurring 38% of the time, was characterized by higher global efficiency, indicating greater integration. Participants engaged with the integrated state more frequently while listening to Webern, whereas they accessed the more segregated state more often during Bach's piece. These findings can be understood within a research framework that views the brain as a dynamic network that continuously reconfigures on both spatial and temporal scales, alternating between integrated and segregated states to adapt to varying environmental and cognitive demands (Alavash et al. 2016; Allen et al. 2014; Betzel et al. 2016; Cole et al. 2013; Sporns 2013; Tognoli and Kelso 2014). The differences in global efficiency and modularity of the states and in the frequency of state occurrence suggest varying processing demands posed by the musical compositions. The greater engagement with the integrated state during Webern's music aligns with evidence that such states arise when higher cognitive effort is required for effective behavioral performance (Kitzbichler et al. 2011). Increased integration, shown to be modulated by task demands (Shine et al. 2016), is hypothesized to facilitate adaptability and cognitive task performance, while reduced modularity has been observed in the presence of greater task demands (Vatansever et al. 2015). Conversely, higher modularity is typically observed in less demanding tasks, indicating less network integration (Cohen and D'Esposito 2016), or more automatic/habitual processing (Shine and Poldrack 2018). Changes in network modularity have also been documented during motor task training (Bassett et al. 2011, 2015). Additionally, the anticorrelation between entropy and the prevalence of the segregated state indicates that as the complexity of the auditory signal increases, the occurrence of the more segregated state decreases, suggesting a connection between the demands of listening conditions and the functional organization of brain states.

In the next step, we investigated expertise‐related differences in whole‐brain organization using graph measures applied to static functional connectivity analysis. Aspiring professional musicians demonstrated significantly higher global efficiency while listening to Webern compared to amateur musicians, indicating a more integrated network configuration that facilitates processing under demanding conditions. This finding aligns with previous research showing that musicians generally exhibit enhanced indices of whole‐brain connectivity, such as degree, density, strength, and global efficiency (Leipold et al. 2021). The absence of expertise‐related differences while listening to Bach suggests that both groups exhibit more similar response patterns to familiar music, in accordance with evidence of higher response similarity in listening to familiar music, regardless of expertise status (Madsen et al. 2019; Oechslin et al. 2013). This indicates that less effortful processing conditions may not require additional functional resources. However, since familiarity with the musical pieces was not assessed, our interpretation is tentative and needs to be tested in future research.

At the same time, the observed expertise‐related differences in global efficiency invite consideration of their potential relevance beyond music perception to more general aspects of cognitive functioning. Prior studies have linked higher network integration to reasoning ability (Hearne et al. 2017) and flexible adaptation to task demands (Kitzbichler et al. 2011; Shine et al. 2016; Vatansever et al. 2015). The literature on music training and cognitive abilities is extensive but mixed, with some studies reporting advantages in musicians (Hao et al. 2023; Zuk et al. 2014) and others finding no such effects (Chen et al. 2022; Gade and Schlemmer 2021; Talamini et al. 2017). In conjunction with evidence suggesting that musical expertise is associated with adaptations extending beyond sensory and motor cortices to higher‐order cognitive regions and cross‐modal integration areas (Jancke 2016), our findings may tentatively be interpreted as reflecting such broader neurocognitive adaptations in aspiring professional musicians.

The analysis of the nodal measures and the investigation of between‐group differences in regions acting as hubs and connector hubs follows up on previous findings showing that groups with different expertise levels utilize different regions as hubs during musical processing (Alluri et al. 2017; Loui et al. 2012). Here, the group of aspiring professional musicians exhibited higher participation and participation coefficient in a wide range of regions, especially during the challenging musical conditions, in comparison to the group of amateur musicians. This indicates greater flexibility in network organization for the group of aspiring professionals, with adaptive changes in functional connectivity and communication among different subnetworks in response to differing demands posed by different listening conditions. Relevant evidence in the literature suggests that flexible hub connectivity patterns facilitate adaptive task performance and that changes in community interactions are modulated by task demands (Cole et al. 2013; Douw et al. 2016).

The regions where aspiring professional musicians exhibited higher participation and participation coefficients in comparison to amateur musicians during listening to Webern are repeatedly reported for their prominent role in auditory processing. Regions of the temporal lobe, like STG, ITG, MTG, and the planum polare, are considered core auditory processing regions in relation to various aspects of musical processing (Koelsch 2011). Differences between musicians and nonmusicians in graph measures, including degree, clustering, and local efficiency in those regions, have also been reported by other studies. Furthermore, activity in the right rSTG, the right inferior frontal gyrus, and the anterior cingulate and paracingulate gyrus, regions here reported as hubs and connector hubs, has been shown to effectively discriminate between musicians and nonmusicians (Saari et al. 2018).

Frontal and parietal regions, including the inferior and middle frontal gyrus, the frontal pole, the parietal and frontal operculum, and the insula, were also prominent in aspiring professionals while listening to Webern. These have been associated with cognitive aspects of musical processing and integration of multisensory information (Quirmbach and Limanowski 2022; Tillmann et al. 2006; Gogolla 2017; Koelsch et al. 2021; Uddin et al. 2017). Also in previous studies, enhanced functional connectivity among frontal, parietal, and auditory regions has been observed in musicians (Tanaka and Kirino 2018; Zamorano et al. 2017). Additionally, the insula, alongside the anterior cingulate cortex, has been reported to exhibit increasing node degree for decreasing onset ages of musical training (Zamorano et al. 2017).

Occipital regions acting as hubs and connector hubs during both pieces for aspiring professionals, namely the temporal occipital fusiform cortex, the anterior fusiform cortex, and the lateral occipital cortex, are considered to be centers of multisensory integration, and have been associated with musical notation reading and processing aspects of musical richness (Satoh et al. 2015). The fusiform gyrus, together with the amygdala and anterior STG, has additionally been reported as a network for emotion‐related processing during music listening (Pehrs et al. 2014). The nucleus accumbens, exhibiting a higher degree for aspiring professionals during Webern, is linked to reward processing and music‐induced pleasantness (Gold et al. 2019; Shany et al. 2019). The caudate nucleus, showing increased degree for professionals during Bach's piece, is involved both in emotion and rhythm processing (Pando‐Naude et al. 2021), rhythmic entrainment (Kokal et al. 2011; Trost et al. 2014) and integration of rhythmic and tonal information (Musacchia et al. 2014).

Altogether, the aspiring professional musicians in comparison to the amateur musicians, engaged brain regions alongside the dorsal and ventral auditory streams as well as higher‐order associative regions, known to partake in perceptual, cognitive, emotional, and reward‐related musical processes, especially in the musically more challenging listening condition, and they additionally engaged only a subset of those regions during the less demanding listening condition. When considered alongside their increased global efficiency—indicating enhanced short‐range and long‐range connectivity and greater network adaptability—these findings may reflect neural network adaptations to intensive musical training. Furthermore, given the musical stimuli chosen, observed differences in network dynamics may reflect adaptation to contextual unpredictability, in line with predictive coding models of music (Koelsch et al. 2019; Vuust et al. 2009). Future studies could specifically investigate how musical training shapes expectation and adaptation mechanisms during naturalistic music listening.

Finally, we would like to address some of the limitations of the current study. A first issue is that the discussed musical features of the pieces are not time‐locked to the neural signals captured by the fMRI. While the quantitative analysis of some features of the musical pieces highlighted some tonal and rhythmic differences, these could not be precisely mapped to neural activity, which is probably affecting the granularity of our findings. It has been argued that key clarity and pulse clarity are among the features that are less reliably extracted by the MIR Toolbox. Lower reliability can reduce the accuracy of observed differences between pieces, so comparisons across measures with varying reliability should be interpreted cautiously—ideally correcting for unreliability if reliability estimates are available (Brandmaier 2025). Additionally, participants' familiarity, exposure, and esthetic preferences for the pieces were not assessed, which could influence the results—especially for the aspiring professionals, who may have had more exposure to contemporary music while attending their preparatory courses, as part of a more elaborate curriculum in music studies. The lack of this information does not allow for more precise interpretation of how observed neural dynamics relate to aspects of music‐induced pleasure, cognitive engagement, or predictive processing mechanisms. Future studies may specifically target how familiarity, exposure, and esthetic appreciation shape participants' processing of the pieces. Furthermore, among aspiring professional musicians, there was a wide variety of primary instruments of practice, which results in inhomogeneity in their expected experience with specific kinds of music. As both groups in our sample consist of individuals with musical backgrounds, the study lacks a baseline comparison with a control group of nonmusicians. Including such a control group in future studies will help clarify how varying levels of musical exposure—including its absence—influence the observed network dynamics.

In relation to the methods applied, DFC analysis requires some parameter choices, like window length or overlap of windows, which are shown to result in variable outcomes, as underlying processes of interest develop on different timescales (Lurie et al. 2019; Preti et al. 2017; Shine and Poldrack 2018). Too short time windows can induce spurious fluctuations and increased noise sensitivity, while too long window sizes can hinder the detection of temporal variations of interest (Preti et al. 2017), and a given window size might not capture reconfigurations of brain networks developing on different time scales (Lurie et al. 2019). We used recommended parameters to ensure robustness, but these decisions inevitably limit the results (Lurie et al. 2019; Preti et al. 2017; Shine and Poldrack 2018). Furthermore, using pairwise Pearson's correlation is only one of several possible ways to uncover relationships between brain regions and does not capture all aspects of functional brain organization (Preti et al. 2017). K‐means clustering used here to uncover states based on DFC is only one of the existing methods, including hierarchical clustering and hidden Markov models, and there is no consensus yet on which one is the optimal choice for different occasions (Preti et al. 2017). In this analysis, subjects were only allowed to be in one specific state at a given point in time, while multiple states might be present at a given point in time to varying degrees. The modularity index, computed here using maximization of the modularity function, partitions a network into a set of communities in a nondeterministic way and produces many near‐optimal partitions of the network (Bassett and Gazzaniga 2011; Sporns and Betzel 2016). This issue was partly addressed by multiple iterations of the algorithm, followed by choosing the most stable partitions.

The thresholding approach we used for the construction of the matrices does not guarantee equal density across participants and groups, which is critical for metrics that depend directly or indirectly on node degree. To further minimize bias, degree and participation coefficient were compared only for nodes that survived thresholding across all participants. Hub detection can be done using numerous different graph measures, most of which express aspects of node centrality, including degree, closeness centrality, eigenvector centrality and betweenness centrality, and not one of them is necessary and sufficient for exhaustive hub detection (van den Heuvel and Sporns 2013) as they might yield slightly different results, although different metrics are often found to be highly correlated (Zhao et al. 2019). In our case, the choice of the measure of degree was to assist and simplify interpretation and is in no way meant as an exhaustive description. Additionally, degree was computed on the whole brain network, and not within each region's community in order to compute within‐group comparisons for the group of aspiring professionals between the two listening conditions.

## 5 Conclusion

In this study, we investigated how two distinct musical pieces—each evoking different processing demands due to their compositional styles—elicit distinct patterns of brain organization during music listening. Listening to the more challenging music piece was associated with a more integrated brain state. Furthermore, the group with higher musical expertise exhibited greater network integration when processing more demanding music and showed greater flexibility in recruiting neural circuits in response to the specific demands of each piece. These results highlight how whole‐brain configurations adapt to varying processing demands and emphasize the role of musical expertise in shaping network dynamics.

## Funding

This work was financially supported by the Max Planck Institute for Human Development and by an intramural grant from the Innovation Fund of President of the Max Planck Society given to UL.

## Conflicts of Interest

The authors declare no conflicts of interest.

## Supporting information


**Data S1:** Supporting Information.

## Data Availability

The data that support the findings of this study are available on request from the corresponding author. The data are not publicly available due to privacy or ethical restrictions.
