# Supplementary material for: Expertise‐Dependent Brain Network Organization During Music Perception
Source: Hum Brain Mapp. 2025 Nov 29;46(17):e70420. doi: 10.1002/hbm.70420 (PMC12663603; doi:10.1002/hbm.70420)
Supplement: Supplementary file 1 — Data S1: Supporting Information. [file HBM-46-e70420-s001.docx]

**Supplementary Information**

**Methods**

Musical feature analysis of the two pieces

Key clarity: In music theory, the key is a group of pitches that forms the basis of a composition. Keys have a tonic note (and corresponding chord), relate uniquely to other pitches of the same or a different key.

Pulse clarity: Pulse clarity is estimated based on tempo, by detecting periodicities in a range of beats per minute (BPMs), and choosing the maximum periodicity score for each defined time frame separately. Periodicities are detected on the detection curve based on the autocorrelation function of the detection curve (Lartillot et al., 2008; Lartillot & Toiviainen, 2007).

Chromagram: The chromagram contains the information of the audio signal, classified in the 12 pitch classes of the equal-tempered scale within an analysis time segment. It depicts the relative intensity of each of the 12 pitch classes in the audio segment chosen. Pitch class refers to a set of pitches that are whole numbers of octaves apart.

The parameters for the estimation of the chromagram, were set to *Min* = 100 Hz, *Max* = 6400 Hz (Gómez, 2006) and *Threshold* = 20 dB.

**Results**

**Figure 1.** Musical pieces analysis by means of automated extraction. **(A)** Chromagram depicting the distribution of energy along pitch classes for the piece by J.S. Bach and **(B)** by A. Webern.

**
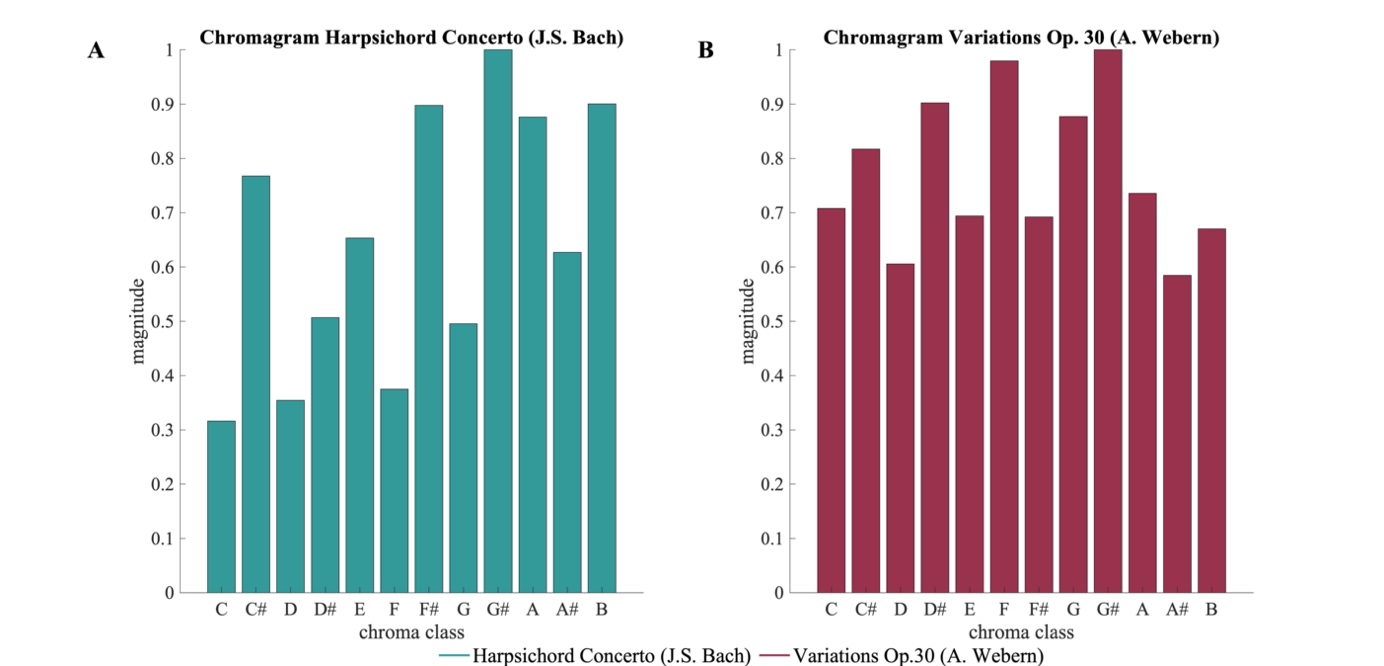
**

**Table 1.** Numerical values of permutation entropy for each segment of both musical pieces.

| **J. S. Bach** | **A.Webern** | **Segment (60s)** |
| --- | --- | --- |
| 0.3098391 | 0.5944348 | 1 |
| 0.3327825 | 0.5678124 | 2 |
| 0.2918116 | 0.5307985 | 3 |
| 0.3023459 | 0.5151746 | 4 |
| 0.3125678 | 0.3946997 | 5 |

**Table 2.** Descriptive statistics of the state metric of frequency computed to characterize participation of individuals in the states across the two listening conditions.

|  | | | **J.S. Bach** | | | | **A. Webern** | |
| --- | --- | --- | --- | --- | --- | --- | --- | --- |
| **State metric** | | | Mean | | Standard deviation | | Mean | Standard deviation |
| frequency state 1 | | | 0.66 | | 0.29 | | 0.57 | 0.2 |
| frequency state 2 | | | 0.33 | | 0.33 | | 0.42 | 0.2 |
|  |  |  | |  | |  |  |  |

Network differences between listening conditions and between groups - Control analysis

To ensure the robustness of our findings, we additionally repeated the analysis assessing network differences between listening conditions and expertise-related differences in whole brain network topology using the Dosenbach 160 atlas (Dosenbach et al., 2010). the dynamic functional connectivity analysis and the k-means clustering resulted again in two most prominent states across listening conditions, State 1 occurring with a mean frequency of 67% and State 2 occurring with a mean frequency 33%. The two states differed significantly in terms of modularity (*F*(1,1) = 4.53, *p* = .035) and global efficiency *F*(1,1) = 5.11, *p* = .03). Post-hoc Tukey-Kramer tests showed that higher modularity values were prevalent in State 1 (*t* = 2.0006, *p* = .04, Cohen's *d* = 0.4; State1, mean = 0.18, SD = 0.15; State 2, mean = 0.16, SD = 0.06), whereas higher global efficiency was more prevalent in State 2 across listening conditions (*t* = 2.3054, *p* = .0023, Cohen's *d* = 0.56; State1, mean = 0.36, SD = 0.05; State 2, mean = 0.39, SD = 0.08). The analysis for the state metric of frequency, which refers to the proportion of time windows participants spent in each state, showed that the State 1 was visited more frequently while listening to Bach (*t* = 2.0425, *p* =:0.04; Bach, mean = 0.711, SD = 0.29; Webern, mean = 0.605, SD = 0.36) and that State 2 was more frequently visited while listening to Webern (*t* = 2.2088, *p* = 0.03; Bach, mean = 0.3, SD = 0.29; Webern, mean = 0.2 , SD = 0.35).

With regards to the static functional connectivity analysis, looking into differences between the two expertise groups, we report again that only during listening to Webern were there group differences in global efficiency (*t =2.4111,* p = 0.0208, Cohen’s d=0.778; aspiring professionals, mean = 0.397, SD = 0.06; amateur musicians, mean = 0.347, SD = 0.06), whereas group differences were not reliable while listening to Bach (*t =*1.0857, p = 0.2846, Cohen’s d=0.353; aspiring professionals, mean = 0.3974, SD = 0.06; amateur musicians, mean = 0.3475, SD = 0.06).

**Table 3.** Brain regions significantly different between the two groups for the nodal measures of degree and participation coefficient for each listening condition. In rows 1-4, the regions for which aspiring professionals had higher degree and participation coefficient values in comparison to the group of amateur musicians. Regions reported at a threshold p < .05, following FDR-correction. In row 5, brain regions that differ significantly between listening conditions within the group of aspiring professionals, for the measure of degree. Higher degree values for these regions occurred during listening to the piece by A. Webern. Again, regions reported at a threshold p < .05, following FDR-correction.

| **condition-**  **group comparison** | **graph measure** | **brain regions** | |
| --- | --- | --- | --- |
| *listening to A. Webern*  aspiring professionals > amateur musicians | degree | frontal lobe  temporal lobe  parietal lobe  occipital lobe  insular lobe  subcortical structures | *right middle frontal gyrus,*  *right frontal operculum,*  *left precentral gyrus,*  *right inferior frontal gyrus-pars triangularis,*  *left & right posterior inferior temporal gyrus,*  *right posterior superior temporal gyrus,*  *left parietal opercular cortex,*  *right posterior supramarginal gyrus,*  *right angular gyrus,*  *left lateral superior occipital cortex,*  *right paracingulate gyrus,*  *left cuneus,*  *left supracalcarine gyrus,*  *left insula,*  *right thalamus,*  *right caudate,*  *left putamen,*  *right accumbens* |
| *listening to A. Webern*  aspiring professionals > amateur musicians | participation coefficient | frontal lobe  temporal lobe  occipital lobe  insular lobe  subcortical structures | *left & right frontal pole,*  *left middle frontal gyrus,*  *left & right inferior frontal gyrus-pars triangularis,*  *right posterior superior temporal gyrus,*  *left posterior inferior temporal gyrus,*  *left planum polare,*  *right temporal fusiform cortex-posterior division,*  *left superior lateral occipital cortex,*  *right inferior lateral occipital cortex,*  *left & right insula,*  *right paracingulate gyrus,*  *right posterior parahippocampal gyrus,*  *right caudate* |
| *listening to J.S Bach*  aspiring professionals > amateur musicians | degree | subcortical structures | *left & right caudate* |
| *listening to J.S Bach*  aspiring professionals > amateur musicians | participation coefficient | frontal lobe  temporal lobe | *right inferior frontal gyrus-pars triangularis,*  *right inferior temporal gyrus-anterior division,*  *right temporal fusiform cortex-anterior division,*  *right temporal occipital fusiform cortex* |
| *listening to A. Webern > listening to J.S. Bach*  aspiring professionals | degree | frontal lobe  temporal lobe | *right inferior frontal gyrus-pars triangularis,*  *right inferior frontal gyrus-pars opercularis,*  *left posterior middle temporal gyrus,*  *left & right temporooccipital middle temporal gyrus,*  *left & right posterior inferior temporal gyrus,*  *right temporooccipital inferior temporal gyrus,*  *right posterior supramarginal gyrus,*  *right temporal fusiform cortex,*  *left temporal occipital fusiform cortex* |
